# Studies of lncRNAs in DNA double strand break repair: what is new?

**DOI:** 10.18632/oncotarget.22090

**Published:** 2017-10-26

**Authors:** Zhenzhen Wu, Yuming Wang

**Affiliations:** ^1^ State Key Laboratory of Ophthalmology, Zhongshan Ophthalmic Center, Sun Yat-Sen University, Guangzhou 510060, China

**Keywords:** DNA double strand break, DNA repair, lncRNA, cancer, chemoresistance

## Abstract

The ‘junk DNA’ that has haunted human genetics for a long time now turns out to hold enormous hidden treasures. As species had their genomes and transcriptomes sequenced, there are an overwhelming number of lncRNA transcripts being reported, however, less than 100 of them have been functionally characterized. DNA damage is recognized and quickly repaired by the cell, with increased expression of numerous genes involved in DNA repair. Most of the time the studies have focused only on proteins involved in these signaling pathways. However, recent studies have implied that lncRNAs can be broadly induced by DNA damage and regulate DNA repair processes by various mechanisms. In this paper, we focus on recent advances in the identification and functional characterization of novel lncRNAs participating in DNA double strand break repair.

## INTRODUCTION

Our genome integrity is under attack every day by a variety of exogenous and endogenous sources [1]. Multiple cellular DNA repair mechanisms exist to remove damaged regions of chromosomes [2]. Besides DNA repair, the genetic information can also be protected by other biological processes such as cell cycle checkpoints and apoptosis [3]. Depending on the damage sources, DNA encounters diverse types of lesions such as base modifications, single strand breaks (SSB), or double strand breaks (DSB) that perturb the primary structure of DNA [4]. Among DNA damage, DSBs are the most deleterious DNA lesions in light of their high levels of propensity to evoke genomic instability and cancer [5]. Homologous recombination (HR) and nonhomologous end joining (NHEJ) are the two major pathways responsible for the repair of DSBs in higher eukaryotes. Mutations in DSB repair proteins are frequently associated with an increased risk of cancer [6]. Besides, hyperactivation of DSB repair genes is one of the reasons for radio- and chemoresistance [3]. In addition to understanding the critical roles of protein-coding driver genes in DNA damage response (DDR), efforts have been focused on identifying long non-coding RNAs (lncRNAs) that are largely transcribed from cancer risk loci and investigating how they can be potential biomarkers during anti-cancer therapy [7]. LncRNAs are deregulated in cancer tissues and their altered expressions are most likely caused by copy number variations or single nucleotide polymorphisms (SNPs) [8, 9]. Given that the majority of lncRNAs have no assigned function, they are likely to provide an abundance of opportunities for revealing novel pathways that could conceivably be targeted for cancer therapy. Throughout this review, we guide readers to the most recent studies that describe in great detail unique characteristics of lncRNAs during DSB repair pathways. We first define the link between DSB and tumorigenesis and then describe noteworthy anti-cancer regimens using DSB repair. Finally, we focus on reviewing the functional roles of lncRNAs in DDR.

### DNA double-strand break repair

DNA double strand breaks are among the most detrimental damages which can lead to severe genome rearrangements. The two main repair pathways triggered by DSBs are homologous recombination and nonhomologous end joining repair [10]. It has been proposed that chromatin state and damaged positions determine which pathway is favored [11, 12]. The error-free homologous recombination repair is a multistep procedure containing three main steps: initially (presynaptic phase), DSB is recognized and processed to give a 3’ single-stranded overhang by the MRE11-RAD50-NBS1 (MRN) complex [13]. This 5′–3′ DNA end resection is reinforced by replication protein A (RPA) [14]. Next (synaptic phase), DNA strand invasion takes place when RAD51 binds to single stranded DNA and displaces RPA, which leads to RAD51 polymerization. RAD52 and p53 can control this process [15]. After the homology search, the heteroduplex structure is formed and stabilized by RAD54/p53 complex [16]. Finally (postsynaptic phase), DNA polymerases use the intact sister chromatid strand to re-synthesize fragments and the Holliday junctions are resolved by specific endonucleases that are called as resolvases [17].

When the homologous template is unavailable, the break ends are directly ligated through nonhomologous end joining repair. The initial step in NHEJ repair is the recognition and binding of the Ku70/80 heterodimer to the DSB [18]. Subsequently, Ku serves as a scaffold to bring the other NHEJ factors to the damage site, including DNA-PKcs, XRCC4, DNA ligase IV, XRCC4-like factor (XLF) and/or ATM and ATR [19]. Interestingly, the order of the sequential recruitment of these factors to the DSB mediated by the Ku heterodimer is quite flexible [20]. Upon binding, Ku-DNA-PKcs or XRCC-XLF can bridge and stabilize DSB ends by protecting them from non-specific processing which may lead to chromosome aberrations. The next step is to make the ends ligatable by removing the damaged or mismatched nucleotides at DSB ends. Many enzymes, including PNKP, Artemis and Ku itself, have been reported responsible for processing DNA ends for the NHEJ pathway [21–23]. The ultimate step in NHEJ is gap filling by DNA polymerase and ligation of the broken ends by DNA Ligase IV whose activity can be stimulated by XRCC4 [24].

### DNA double-strand break repair, tumorigenesis, and drug resistance in cancer

DNA double-strand break repair pathways have a multifaceted function in tumorigenesis and in the response to therapeutic modalities. Firstly, erroneous or deregulated DNA repair results in chromosomal abnormalities, genomic instability, and higher mutation rates, which can predispose the cells to cancer and make them vulnerable to certain kinds of genotoxic stresses [25]. On the other hand, hyperactive DNA repair proteins due to upregulation or polymorphisms may provide survival advantages to cancer cells in therapeutic response [26]. Defects in core HR and NHEJ proteins have been implicated in a vast repertoire of cancers (Table 1). It has been estimated that approximately half of high-grade serous ovarian adenocarcinoma samples are defective in HR repair pathway, and these HR defects are largely driven by mutations or epigenetic silencing of BRCA1 and BRCA2 genes [27]. In terms of the major cellular sources of DSBs, evidence has shown that the DDR can be invoked and dysfunctional at an early stage in the development of neoplasia [28, 29]. The activation of oncogenes, for example, MYC and RAS can stimulate the firing of various unwanted replication forks as a major aspect of a proliferative program. These forks rapidly stall and collapse, resulting in formation of DSBs [30, 31]. Cell-cycle checkpoints are elicited to repair DNA lesions before mitosis takes place. For precancerous damage to advance to tumors, it is suggested that DSB repair factors and cell-cycle checkpoint proteins progress toward becoming inactivated. Thus, cells continue through the cell cycle with unsuccessfully repaired collapsed forks in place, resulting in tumor growth and expansion [32]. Additionally, there is strong association between DSB repair gene mutations and an elevated risk of inherited rare diseases. Mutations in ATM, Mre11 and NBS1 are found in patients with Ataxia Telangiectasia (A-T), Ataxia Telangiectasia-like disorder (A-TLD) and Nijmegen breakage syndrome (NBS), respectively [33].

**Table 1:** Associated defects of HR and NHEJ proteins in various cancers

Proteins	Cancer types	Ref
HR		
RAD51	breast cancer, pancreatic cancer, head and neck squamous cancer, soft tissue sarcoma	[77–81]
RAD50	breast carcinoma, melanoma, ovarian cancer, colorectal cancer, head and neck squamous cancer	[82–86]
MRN complex		
CtIP	breast cancer	[87]
RPA	colon cancer	[88]
RECQL5	breast cancer	[89]
RTEL1	lung cancer, gastrointestinal tract tumors	[90, 91]
HR/NHEJ		
BRCA1/BRCA2	breast cancer, ovarian cancer	[92, 93]
FA pathway proteins	Fanconi anemia	[94]
XRCC1	prostate cancer, bladder cancer, head and neck cancer	[95–97]
POLQ	breast cancer, ovarian cancer	[98]
NHEJ		
Ku70/80	gastric cancer, breast cancer	[99, 100]
DNA-PKcs	gastric cancer, breast cancer, oral squamous cell carcinoma, lung carcinoma, esophageal cancer	[101–105]
Artemis	Colorectal cancer, breast cancer, lymphoid cancer	[106–108]
WRN	Colorectal cancer	[109]
Ligase IV/XRCC4	pediatric brain tumor, breast cancer, ovarian cancer	[110–112]

Radiation therapy which causes a variety of DNA lesions including DSBs damage continues to be a mainstay in the treatment of an assortment of malignancies [34]. However, tumor cells as seen in many cancers often display resistance to standardized radiation therapy due to hyperactive DSB repair mechanisms [35]. Therefore, developing drugs aimed at modulating DSB repair activity has provided a profound avenue for many commonly used chemotherapy and radiotherapy regimens. One of the well-known cases is the utilization of platinum salts which is frequently given in patients with advanced ovarian cancer [36]. Platinum salts (carboplatin or cisplatin) can cause DNA inter- and intrastrand crosslinks damages that are recognized and repaired by a combination of nucleotide excision repair (NER) and HR [37]. It has been estimated that nearly half of high-grade serous ovarian cancers have germ line or somatic mutations in BRCA1 or BRCA2 [38, 39]. Many DSB repair proteins are now being used as biomarkers to direct the use of therapy (Table 2). Although tumor cells defective in the repair genes show sensitivity toward genotoxic agents in the first place, after an unpredictable period, hyperactivity of the repair proteins due to re-emergence, reversal or overlapping compensatory pathways can make cancer cells resistant and account for the relapse [3]. This is especially true also in ovarian cancer, in which more than 42% of the carboplatin-resistant tumors tested had secondary mutations that restored the BRCA1 or BRCA2 open reading frames [40]. Tumor cells are highly heterogenous and have the ability to develop either intrinsic or acquired resistance phenotype through molecular alterations. This poses a major challenge to cancer treatment. One possible way to overcome or delay the development of chemoresistance is to ‘re-sensitize’ tumors to the original treatment with the help of new identified targets.

**Table 2:** Potent inhibitors of DSB repair in clinical use and development

Targets	Inhibitors	Stage of development	Cancer type	Ref
DNA-PKcs	CC-115	Phase I clinical trial	Myeloma, non-Hodgkin lymphoma	[113]
	MSC2490484A			
	CC-122			
ATR	AZD6738	Phase I clinical trial	Various tumors	[114, 115]
	VX-970	Phase I and phase II clinical trials	Solid tumor, relapsed small cell lung cancer	[116]
ATM	AZD0156	Phase I clinical trial	Advanced tumors	[117]
CHK1	MK-8776	Phase I clinical trial	Acute leukemia, advanced solid tumors	[118, 119]
CHK1/2	CBP501	Phase I clinical trial	Advanced solid tumors	[120]
		Phase II clinical trial	Malignant pleural mesothelioma	[121]
	AZD7762	Phase I clinical trial	Advanced solid tumors	[122]
PARP	BMN673	Phase I clinical trial	Advanced solid tumors	[123]
	Olaparib	Phase I clinical trial	Glioblastoma, advanced solid tumors	
	AZD2281	Phase I clinical trial	Triple negative breast cancer, ovarian cancer	
	Niraparib	Phase I clinical trial	Ewing's sarcoma	
	Veliparib	Phase I clinical trial	Triple negative breast cancer, ovarian cancer	

### LncRNAs, DSB, and cancer

Besides DSB repair proteins, mutations in long non-coding RNAs (lncRNAs) are associated with tumorigenesis. Large scale screening has provided novel p53 interactors, including lncRNAs, which can be potential therapeutic targets [41–43]. Researchers have identified 22 distinct lncRNAs that are involved in the regulation of chemoresistance in cancers [44]. How the lncRNA regulatory networks act in concert to modulate oncogenesis and therapeutic response remains largely unknown. Here we review the most recent findings on lncRNAs with well-characterized functions in DDR.

### DINO – the p53 stabilizer

**Figure 1:**
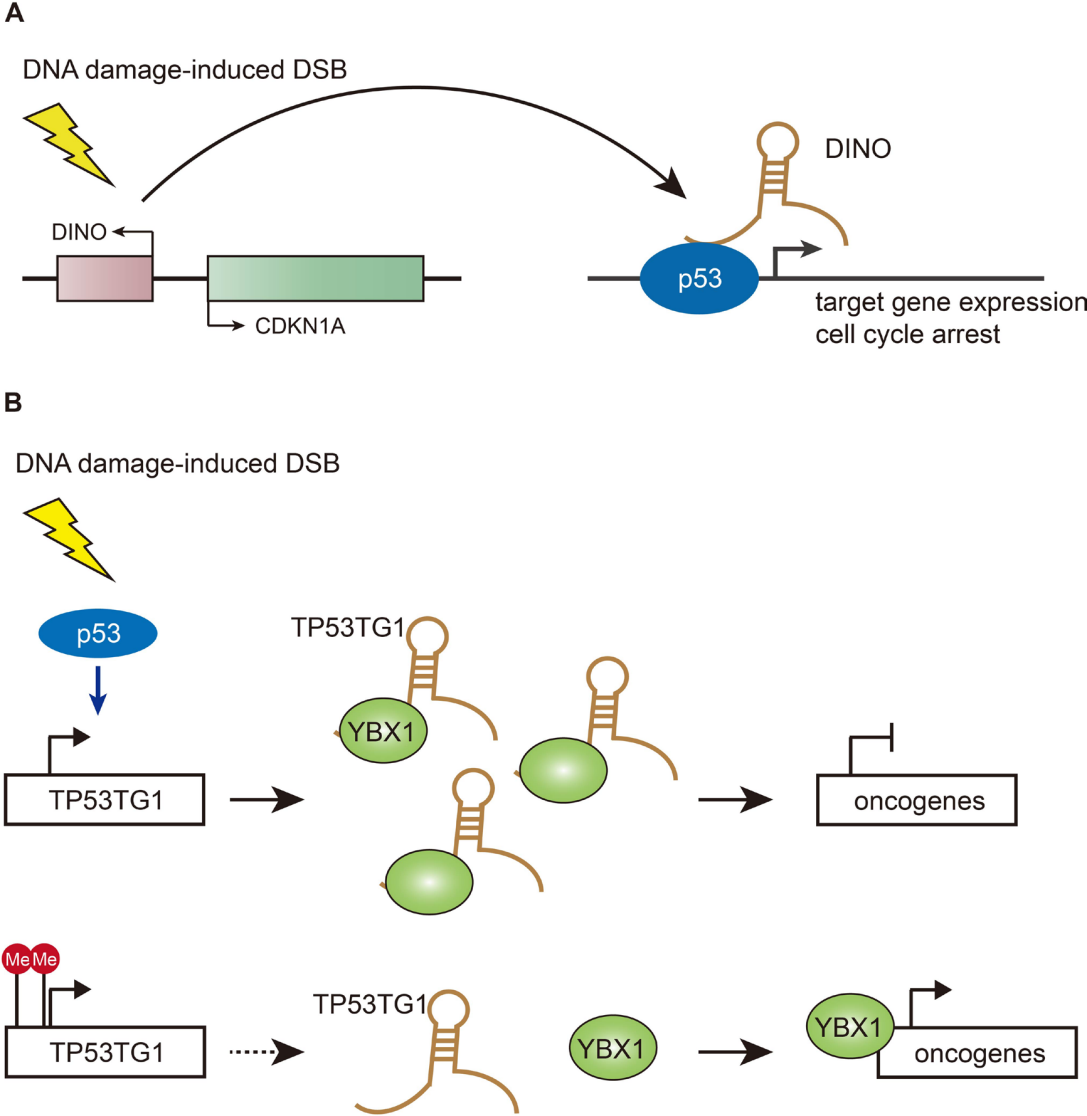
Functions of DINO and TP53TG1 in DSB repair pathways (**A**) DINO is increased upon DNA damage in a p53-dependent manner and physically interacts with p53, resulting in p53 stabilization and activation of p53 target genes cell cycle arrest. (**B**) TP53TG1 is stimulated by p53 upon DNA damage and binds to the DNA/RNA binding protein YBX1 to prevent its activation of oncogenes. TP53TG1 inactivation by methylation in cancer cells releases the transcriptional repression of YBX1-targeted growth-promoting genes.

The fast-growing RNA-seq technique has found that distinct sets of lncRNAs are expressed in correlation with different physiological and pathological cellular processes [45]. The earliest attempts to elucidate the function of thousands of lncRNAs with highly conserved chromatin signatures in specific biological pathways have shown that 39 lncRNAs were significantly induced in p53-mediated DNA damage response [46]. Most recently, Howard Chang and colleagues performed a screen for transcribed regions around the promoters of cell cycle genes and discovered a new regulatory lncRNA – DINO (damage-induced non-coding RNA), expanding the p53 network (Figure 1A) [41, 47]. DINO is transcribed divergently from CDKN1A (p21) promoter, with ~100-fold increase upon doxorubicin treatment in a p53-dependent manner [47]. In terms of the functions of DINO in the p53-mediated DNA damage pathway, the authors have found that DINO can physically interact with the C-terminal RNA-binding region of p53 and colocalize at multiple p53 target genes including CDKN1A throughout the genome to co-regulate the p53-dependent gene expression and cell cycle arrest in response to DNA damage. More importantly, the microhomology region of DINO interacting with p53 is highly conserved in mammal species, although DINO exhibits poor overall sequence identity across species, suggesting that DINO represent a conserved transcriptional response after DNA damage [47]. To further discern whether the effects observed are due to disruption of the DINO transcripts or the DNA binding platform for other regulatory factors, the study presented two different transgenic knockout mouse models in which the promoter of *Dino* is either intact or inactivated. Both lines of *Dino* knockout mice exhibit impaired response to doxorubicin, suggesting that in mouse *Dino* acts in trans similarly to human DINO [47]. This study identified a new lncRNA that constitutes a feed-forward feedback loop in the p53-dependent DNA damage response. However, it remains unclear how DINO stabilizes p53 and whether DINO mutations are putatively correlated with cancer diagnosis and prognosis.

### TP53TG1 – Chemo-sensitizer

Innate and acquired chemoresistance exhibited by most tumors exposed to conventional chemotherapeutic agents account for most relapse cases in cancer patients [48]. In addition to multiple key well-known molecular players, lncRNAs have been shown to be involved in the development of chemoresistance [49]. Most recently, Manel Esteller's group has discovered the lncRNA – TP53TG1 as a chemo-sensitizer to promote p53 response to DNA damage (Figure 1B) [50]. By comparing DNA methylation profiles of colon cancer cell line HCT-116 with or without disrupted DNA methyltransferase enzymes, along with normal colon cell line, the authors have identified a dozen of lncRNAs which exhibited CpG island hypermethylation-associated transcriptional silencing in colon cancer cells. In normal colon tissues, the p53 protein binds the regulatory region of the TP53TG1 molecule and activates it in response to cellular stress [50]. Thereafter, TP53TG1 blocks activation of the YBX1 protein that, when induced, goes into the cell nucleus and stimulates many oncogenes [51]. 10% of colon and stomach tumors show inactivation of the TP53TG1 molecule due to hypermethylation at CpG island, furthermore, oncology patients with inactive TP53TG1 have a shorter progression-free survival [50]. TP53TG1 silencing in cancer cells causes the p53 protein to lose its antitumor effects and free the RNA binding protein YBX1 to activate oncogenes that prevent the death of malignant cells in response to anti-tumor drugs, resulting in chemoresistance [50]. Of course, YBX1 should not be the only ‘hit’ resulting from the epigenetic loss of TP53TG1 in the center p53 link. Alternative targets would warrant further research. Esteller's lab and others have underscored that epigenetic factors are highly associated with multi-resistance of tumors to most common drugs, although it remains unclear how these epigenetic mutations occur in tumor cells.

### NEAT1 – tumor suppressor becoming cancer protector

**Figure 2:**
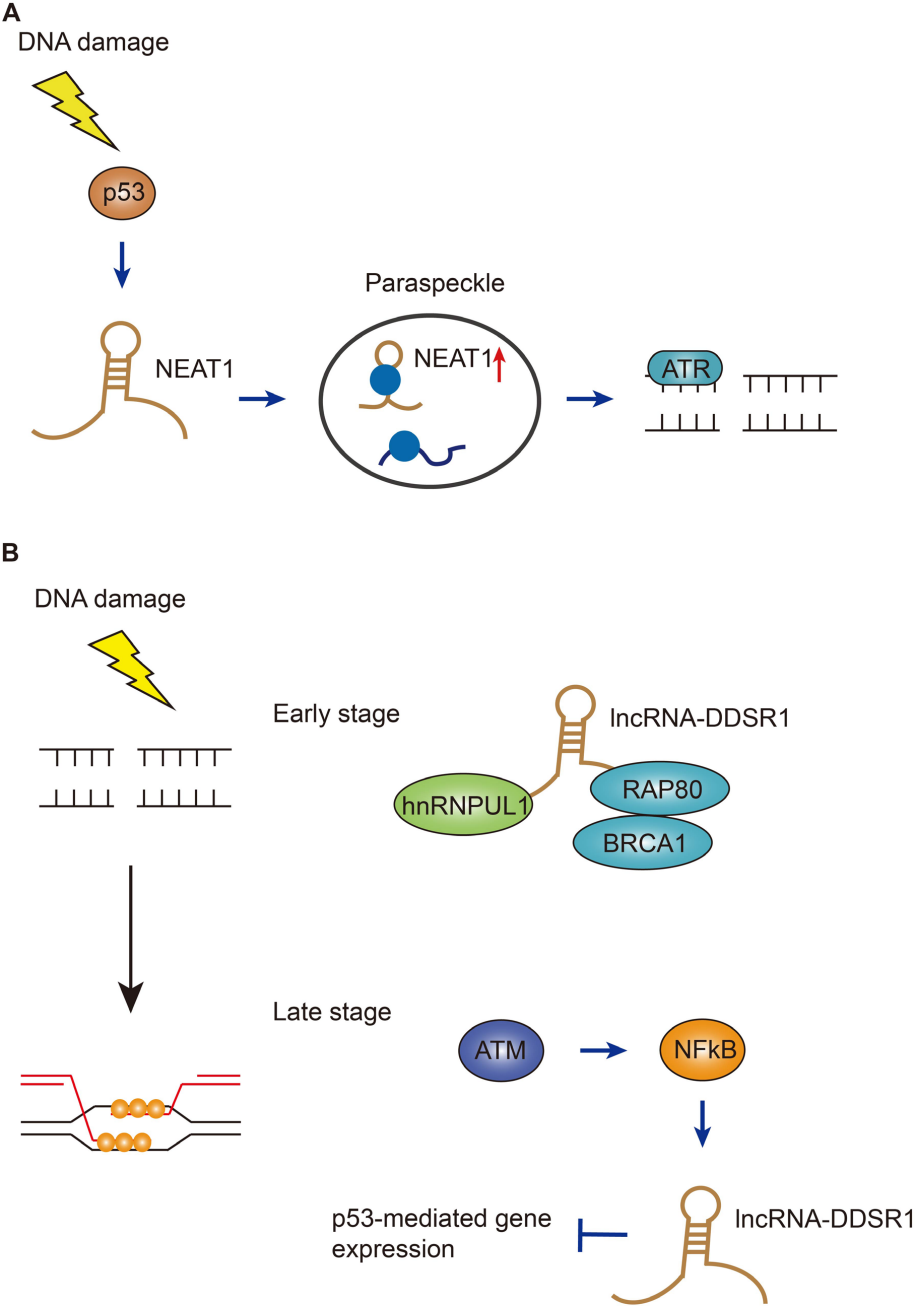
Functions of NEAT1 and DDSR1 in DSB repair pathways (**A**) NEAT1, mainly localized in paraspeckles, is induced by p53 after DNA damage, the paraspeckles with increased NEAT1 then regulates the ATR-mediated DSB repair. (**B**) At early stage of DSB repair, DDSR1 interacts with BRCA1-RAP80 and hnRNPUL1 to prevent them from promiscuous DNA binding; at late stage of DSB repair, DDSR1 is induced by ATM and NF-κB signaling pathways to ensure efficient repair.

Given the vital role p53 plays in cancer, efforts have focused on finding a means of restoring functional p53 in human cancer cells. Normally lncRNAs exert a diverse spectrum of regulatory mechanisms, a special lncRNA NEAT1 (nuclear-enriched autosomal transcript) is mainly localized to nuclear paraspeckles, subnuclear particles that can be found in the cell nuclei of cancer cells [52]. A recent study has illustrated that targeting NEAT1 and ‘paraspeckles’ would be a new therapeutic avenue in the fight against cancer (Figure 2A) [53]. The authors have observed that NEAT1 is increased in Nutlin-3a-treated p53 wild type cancer cell lines. Strikingly, although NEAT1 is regulated by p53, it is required for the survival of highly dividing cancer initiating cells and that mice lacking NEAT1 are protected from developing skin cancer [53]. NEAT1 depleted cancer cells exhibited a much higher level of γH2A.X accumulation and this higher amount of DNA damage was exacerbated in response to replication stress [53]. These surprising results suggest that cancer cells can ‘hijack’ the survival principle of NEAT1 for their own good. However, there is still a long way to go before the information can be harnessed to help cure cancer. For instance, how exactly NEAT1 confers its survival functions to cells is worth further investigation. Interestingly, the most recent study has found that some NEAT1 isoforms reside in numerous non-paraspeckle foci and exert distinct functions [54]. Therefore, precise disruption of NEAT1 isoforms via genome editing tools is demanded in clinical trials for targeting overlapping transcripts.

### DDSR1 – dual roles at early and later stages to orchestrate DSB repair

The non-homologous end-joining (NHEJ) pathway and homologous recombination (HR) pathway share the duty to safeguard the genome stability when the most toxic DNA double-strand breaks occur. It is a crucial decision for cells to choose which pathway to orchestrate, in that making a wrong choice can lead to detrimental repair outcomes [55]. Therefore, efforts are taken to identify the cellular determinants involved in the regulation of these two pathways. In a recent study, Misteli and colleagues treated immortalized human fibroblasts with DSB-inducing agents and found a lncRNA named DDSR1 (DNA damage-sensitive RNA1) which was highly induced upon damage in an ATM and NF-κB dependent manner (Figure 2B) [56]. Interestingly, p53 was not required for DDSR1 induction after DNA damage, but DDSR1 can largely regulate p53 target genes under stress conditions. Moreover, cell proliferation and DNA damage signaling were reduced in cells lacking DDSR1 [56]. In order to investigate the underlying mechanism by which DDSR1 affects DNA repair, the authors applied DSB repair reporter cell lines and found that only the HR pathway was compromised in DDSR1-deficient cells. The authors also found a RNA binding protein hnRNPUL1 physically associating with DDSR1. HnRNPUL1 has been reported to promote DNA end resection in HR pathway [57]. Thus, further analysis demonstrated that depletion of DDSR1 increased accumulation of BRCA1/RAP80 complex at sites of DNA damage to restrict DNA end resection [56]. Given that induction of DDSR1 took several hours to occur, while recruitment of BRCA1/RAP80 to DNA damage sites happens within several minutes, DDSR1 could possibly have dual roles in regulating HR: at early stage, DDSR1 associates with BRCA1/RAP80 complex to prevent them binding to damaged chromatin. Subsequently, DDSR1 expression is increased by ATM and NF-κB to inhibit p53 target gene expression [56]. This study highlights the multifaceted nature of lncRNAs in maintaining genome integrity, providing new insights onto the precise targeted therapy for cancer.

### CUPID1, CUPID2 – co-players to cancer risk factors

**Figure 3:**
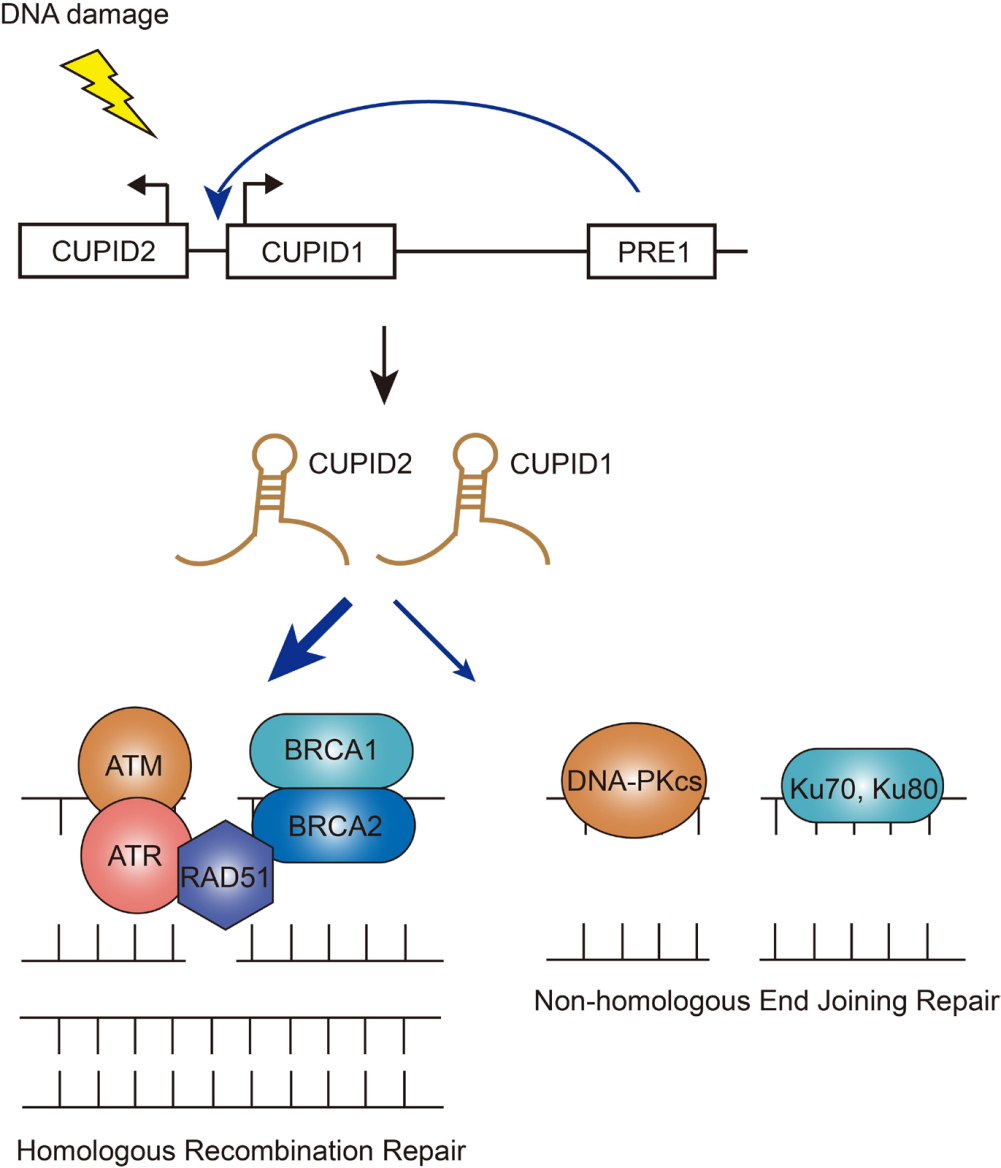
Functions of CUPID1/CUPID2 in DSB repair pathways The two lncRNAs CUPID1 and CUPID2 are transcribed from a bidirectional promoter and regulated by enhancer PRE1. Upon DNA damage, CUPID1/CUPID2 favors a switch from NHEJ to HR DSB repair.

It is always a big challenge to interpret the mechanisms of action of risk-associated SNPs from GWAS analysis, given that more and more SNPs are found to lie in non-coding regions of the genome. Most recently, researchers have identified two novel lncRNAs that were transcribed in the proximal cancer risk loci and characterized the important roles in tumorigenesis (Figure 3) [58]. Previously it's been found by the same group that the strongest risk-associated SNPs fall within the enhancer region named PRE1 that regulates the expression of CCND1 [59]. PRE1 also acts as an enhancer on the lncRNAs CUPID1 and CUPID2 which are transcribed from a bidirectional promoter. The two lncRNAs were highly expressed in breast cancer cell lines dependent on estrogen [58]. However, this induction was not associated with PRE1 region amplification, indicating that copy-number variation is not the only mechanism underlying the expression. Unlike CCND1, silencing of CUPID1 or CUPID2 did not affect cell cycle, although depletion of these two lncRNAs indeed caused deregulation of DNA replication, recombination and repair genes [58]. The authors have illustrated that CUPID1 and CUPID2 can facilitate the formation of phosphorylated RPA foci and promote RAD51 recruitment to DSBs during the initiation step of the HR pathway. Interestingly, when breast cancer risk SNPs were incorporated into PRE1 region, leading to the decreased expression of these two lncRNAs, the overall DSB repair was not impeded, but a large number of structural variants across the genome were observed [58]. These data proposed a clear regulatory role for these two lncRNAs: CUPID1 and CUPID2 prevent the breast tumors from error introduction in response to radiotherapy by favoring a switch from NHEJ to HR DSB repair.

### LINP1 – scaffolding NHEJ repair complex

**Figure 4:**
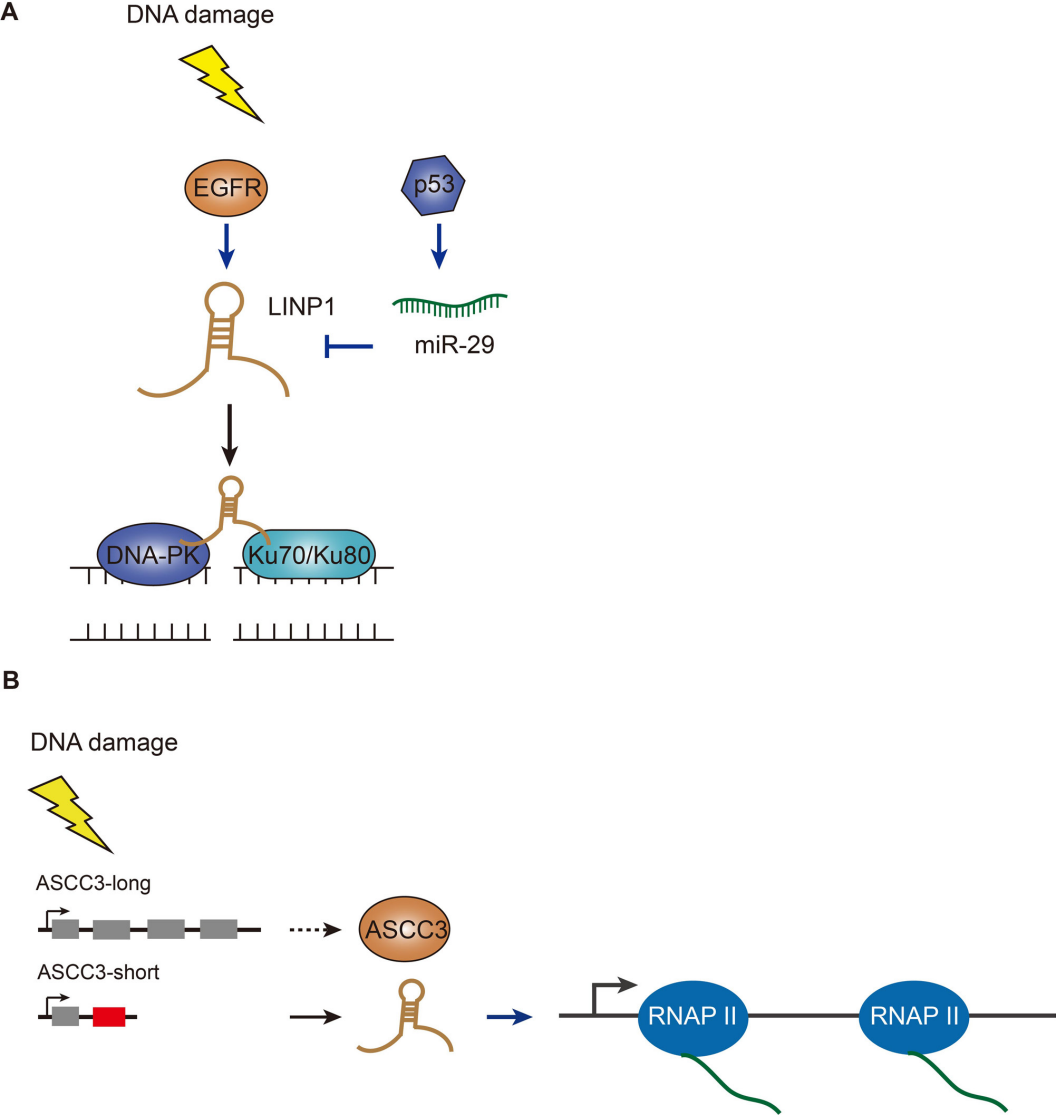
Functions of LINP1 and ASCC3 in DSB repair pathways (**A**) LINP1 is induced by EGFR upon DNA damage and further stabilized by inactivation of miR-29 which is stimulated by p53. LINP1 can physically interact with Ku80-DNA-PKcs complex and promote DSB repair. (**B**) Short isoform of ASCC3 lncRNA is produced via alternative splicing after DNA damage and facilitates transcription recovery after DNA repair; while the long version of ASCC3 (protein coding transcript) inhibits transcription recovery.

The discovery of lncRNAs has dramatically changed the understanding of the biology of human diseases, especially when genomic studies of tough-to-treat cancers have mainly focused on protein-coding genes and provided no effective targeted therapies. Many TNBC (triple negative breast cancer) patients poorly respond to chemotherapy and radiotherapy due to EGFR (epidermal growth factor receptor) gene amplification and TP53 mutations [60, 61]. Most recently, researchers have identified a lncRNA – LINP1 – that regulates the sensitivity of the tumor cells to radiation therapy (Figure 4A) [62]. LINP1 was initially identified as overexpressed in TNBC when compared with other breast cancer subtypes using RNA-seq data from the Cancer Genome Atlas and the Cancer Cell Line Encyclopedia. Among the dozens of lncRNA candidates, LINP1 stands out as functional screening has revealed that LINP1 knockdown enhanced apoptosis in TNBC cell lines following doxorubicin treatment (a chemotherapy drug for TNBC) [62]. By applying *in-vitro* synthesized and also endogenous RNA, the authors demonstrated that LINP1 transcript physically interacted with Ku80-DNA-PKcs complex. Therefore, LINP1 knockdown in TNBC cells led to reduced DSB repair, and conversely, overexpression of LINP1 in ER-positive cells increased NHEJ activity [62]. The authors uncovered that EGFR activation upregulates LINP1 transcription, thus in turn stabilizes the Ku80-DNA-PKcs interaction. On the other hand, TP53 activation stimulates miR-29 that targets LINP1 and down-regulates its expression later point after damage. Thus, TP53 mutations in TNBC would further increase LINP1 expression at the post-transcriptional levels after DNA damage. Given that inhibition of the NHEJ pathway has been proposed by oncology researchers to synergize DNA-damaging therapies for better treatment outcomes for TNBC, LINP1, as a new class of cancer-driver gene that links two repair scaffold proteins, may serve as a novel therapeutic target for TNBC treatment. While this study mainly focused on TNBC, these findings have left an open question: to what extent cellular LINP1 expression levels indicate NHEJ functional status, since overexpression of LINP1 increases resistance to genotoxic insults and loss of expression of LINP1 may impair genome stability.

### ASCC3 short isoform – helps cells recover from DNA damage

Ultraviolet light can damage DNA, triggering a general transient shutdown of gene transcription [63]. This response has been known for a few decades. However, the molecular mechanism underlying transcription shutdown and recovery upon DNA damage is still largely unknown. Also, in contrast to global transcription repression, some genes are activated by UV light [64]. An investigation of this counter-intuitive behavior implies a surprising gene regulation mechanism [65]. The latest studies have focused on identifying novel factors associated with transcription-related changes after UV-induced DNA damage (Figure 4B) [66]. By using next-generation DNA and RNA sequencing technology, the authors have revealed a global switch in pre-mRNA processing resulting in a preference for the production of transcripts containing alternative last exons which are not normally included in the dominant canonical mRNA isoforms. By combining siRNA-mediated functional screening, ASCC3 stands out as a pivotal regulator of transcription following UV damage. Knocking down the short isoform of ASCC3 transcript (functionally a lncRNA) prevented the cells from recovering normal levels of transcription. In contrast, blocking the long isoform of ASCC3 transcript (encoding a full-length protein) increased transcription levels after UV irradiation [66]. This alternative last exon-derived non-coding RNA produced from a protein-coding gene provides a new source of lncRNAs. Too much exposure to UV radiation is the main cause of skin cancer development. A clearer understanding of UV-induced DNA damage repair is crucial in the prevention of skin cancer.

### Outlook and challenges

The simplest definition for lncRNAs is RNA genes larger than 200 base-pairs that do not appear to have coding potential. However, the characteristics of lncRNAs are far more complex than were originally imagined, as they are involved in numerous biological processes across many aspects of life rather than just results of transcriptional noise. In normal proliferating cells, lncRNAs are expressed, on average, at much lower levels than coding genes [67, 68]. During DNA damage response, many lncRNAs are dramatically induced. With current deep sequencing technology, the rate of discovering new lncRNA genes is rapidly overwhelming the rate of characterizing them. The hurdles to characterize lncRNAs are not only due to experimental challenges, but also and more importantly due to ambiguous results from only RNA-seq experiments, as for most lncRNAs, the action of transcription alone is sufficient for their function but the transcript itself is not necessary [69–71]. To discover more functional lncRNAs during cellular response, efforts are still needed to apply multiple powerful approaches. Firstly, given that the biogenesis and processing of lncRNAs is quite distinguished from mRNAs in normal proliferating cells [72], it is likely that the transcriptional profiles of lncRNAs in response to DNA damage have unique features. mNET-seq (mammalian native elongating transcript sequencing) can provide critical evidence on the active transcription of lncRNAs under stress conditions [72]. Secondly, visualizing the mobility of DNA repair factors in real time during cellular responses can be achieved by using local irradiation and live cell imaging [73]. lncRNAs with essential roles in DDR (DNA damage response) signaling pathways can also be detected by using these molecular analyses. Thirdly, unbiased genome-wide CRISPR screening will yield a great appreciation for lncRNAs’ biological functions. Jonathan Weissman's and Daniel Lim's labs have developed a CRISPR interference platform that targeted thousands of lncRNA loci and found hundreds of them for robust cell growth in at least one cell type [74]. This modified CRISPR approach can be expanded to pinpoint lncRNA transcripts that are important during DDR. Lastly, unlike mRNAs, lncRNAs are mostly restricted to nucleus and ~60% of annotated lncRNAs are chromatin-enriched [75], and they are poorly co-transcriptionally processed and are rapidly degraded by the RNA exosome [72]. Due to diverse functions of lncRNAs in various biological phenomena, it would be important to study the localization of these molecules during DDR.

LncRNAs can have pro-survival or pro-apoptotic functions in response to DNA damage that can be utilized for future translational research. Given that expressions and functions of lncRNAs are highly cell type specific, cancer treatments may benefit from targeting lncRNAs crucial to cancer cell function, whereas having little effect on nearby normal cells that do not require these lncRNAs. The connection between DSB repair and ionizing radiation has been solidly established and work to date proposes that targeting the DSB repair pathways still has extensive potential for expanding radio- and chemosensitization in the clinic [76]. Although there have been no efforts so far to develop drugs targeting lncRNAs in a clinical setting, it is encouraging to see that more and more lncRNAs have been identified with well-defined functions, these findings demonstrate that the therapeutic potential of lncRNAs warrants further investigation.
